# Targeted community based interventions improved malaria management competencies in rural Ghana

**DOI:** 10.1186/s41256-017-0048-5

**Published:** 2017-10-02

**Authors:** Isabella A. Quakyi, George O. Adjei, David J. Sullivan, Judith K. Stephens, Amos Laar, Vivian N. Ama Aubyn, Richmond Owusu, Kwame S. Sakyi, Nathaniel Coleman, Francis D. Krampa, Linda Vanotoo, Julliette Tuakli, Bernard B. Bortei, Edward Essuman, Felix Sorvor, Isaac A. Boateng, Constance Bart-Plange, Ebenezer A. Addison, Peter Winch, Andrew A. Adjei

**Affiliations:** 1 Department of Biological, Environmental and Occupational Health Sciences, School of Public Health, College of Health Sciences, University of Ghana, P.O. Box LG 13, Legon, Accra, Ghana; 2 Office of Research, Innovation and Development, University of Ghana, Legon, Accra, Ghana; 3 Department of Molecular Microbiology and Immunology, Johns Hopkins Bloomberg School of Public Health, 615 N. Wolfe St, Baltimore, MD 21205 USA; 4 Department of Population, Family, and Reproductive Health, School of Public Health, University of Ghana, Legon, Accra, Ghana; 5 National Malaria Control Programme, Ministry of Health, Accra, Ghana; 6 Department of Health Policy, Planning and Management, School of Public Health, University of Ghana, Legon, Accra, Ghana; 7 Department of International Health, Social and Behavioural Interventions Program, Johns Hopkins Bloomberg School of Public Health, 615 N. Wolfe St, Baltimore, MD 21205 USA; 8 Department of Biochemistry, Cell and Molecular Biology, University of Ghana, Legon, Accra, Ghana; 9 Regional Health Directorate, Ghana Health Services, Accra, Ghana; 10 Child and Associates, Accra, Ghana; 11 Asante-Akim Central Municipal Health Directorate, Ghana Health Services, Konongo, Ghana; 12 Kpone Katamanso District Health Directorate, Ghana Health Services, Kpone, Tema, Ghana; 13 Worldwide Universities Network, University of Ghana, P.O. Box LG 13, Legon, Accra, Ghana

## Abstract

**Background:**

Malaria is one of the most challenging public health concerns in the developing world. To address its impact in endemic regions, several interventions are implemented by stakeholders. The Affordable Medicine Facility-malaria (AMFm) is an example of such interventions. Its activities include communication interventions to enhance the knowledge of caregivers of children under five years, licensed chemical sellers (LCS) and prescribers on malaria management with artemisinin-based combination therapy (ACT). This study was conducted to evaluate the effectiveness of the AMFm activities on malaria among targeted groups in two rural communities in Ghana.

**Methods:**

A communication intervention study was conducted in the Asante-Akim North and South Districts of Ghana. Repeated cross-sectional pre and post surveys were deployed. Relevant malaria messages were designed and used to develop the information, education and communication (IEC) tools for the intervention. With the aid of posters and flipcharts developed by our study, community health workers (CHWs), prescribers, and licenced chemical sellers provided proper counselling to clients on malaria management. Trained CHWs and community based volunteers educated caregivers of children under five years on malaria management at their homes and at public gatherings such as churches, mosques, schools. Chi-square tests and logistic regression were run to determine associations and control for demographic differences respectively.

**Results:**

There was significantly high exposure to malaria/ACT interventions in the intervention district than in the comparison district (OR = 16.02; 95% CI = 7.88–32.55) and same for malaria/ACT-related knowledge (OR = 3.63; 95% CI = 2.52–5.23). The participants in the intervention district were also more knowledgeable about correct administration of dispersible drug for children <5 years than their counterparts in the unexposed district.

**Conclusion:**

Our data show that targeted interventions improve malaria based competences in rural community settings. The availability of subsidized ACTs and the intensity of the communication campaigns contributed to the AMFm-related awareness, improved knowledge on malaria/ACTs and management practices.

## Background

Malaria is one of the most challenging public health concerns in the developing world. In 2016, it was estimated that 3.4 billion people were at risk of acquiring malaria with populations living in sub-Saharan Africa most affected [1]. Of the above estimated risk, 90% of the cases and 92% of deaths occurred in the WHO African Region with children under five years of age and pregnant women bearing the most brunt [1]. Malaria continues to be a major public health challenge in Ghana, as the leading cause of morbidity and mortality. For instance, in 2016, the country recorded approximately 10.4 million suspected malaria cases representing about 39% of outpatient department (OPD) cases seen; with about 25% and 4% of total admission and total deaths respectively being attributable to malaria [2]. The National Malaria Control Programme (NMCP) is the government agency responsible for prevention and control of malaria in Ghana. The main prevention and control measures currently being implemented include promotion of long-lasting insecticide treated nets (LLINs) through universal coverage; indoor residual spraying (IRS); intermittent presumptive treatment for pregnant women with Sulfadoxine-Pyrimethamine (SP); procurement and distribution of effective anti-malarial drugs according to national policy; seasonal malaria chemo-prevention; training of personnel of the Ghana Health Service (GHS) in malaria diagnosis and management, education at the community level on preventive measures, early detection of symptoms and prompt treatment of malaria [2–4]. There have been positive outcomes for many of these interventions over the years including reduction in malaria morbidity and mortality [5–8].

To sustain the gains in reducing malaria-related morbidity and mortality, it was imperative to replace ineffective anti-malarial monotherapies such as chloroquine whose efficacy had reduced below 50% in Ghana [9–11] with artemisinin-based combination therapy (ACTs). In Ghana, ACTs were made available as over-the-counter medicines in 2004–2005 and this facilitated participation in the implementation of the Global Fund’s Affordable Medicines Facility-malaria (AMFm) initiative. The AMFm initiative which started in 2010, made a payment of 90–95% of the post-negotiation price (the co-payment) on behalf of eligible first-line buyers from the public, private for-profit and private not-for-profit sectors, who purchased ACTs directly from the manufacturers in order to increase access to affordable ACTs through all existing channels of distribution. All ACTs that were procured through the AMFm program had a Green Leaf Logo on the package.

The NMCP of the Ghana Health Service procured these AMFm-ACTs and distributed them through the facilities of the GHS, Licensed Chemical Sellers (LCS) who sell over-the-counter medicines and pharmacies. The NMCP also provided training to the LCS on the treatment of malaria with ACTs and the significance of the AMFm logo. In addition, under the AMFm supporting interventions, early and appropriate treatment with ACTs were further promoted through television advertisement, posters and other media. Previously, Minja et al. [12] have assessed theory driven social and behavioural change communication for insecticide treated nets use in Tanzania. The role of communication between clients and health care providers has also been demonstrated to have implications for adherence to malaria treatment [13]. More recently, Koenker et al. [14] demonstrated the strategic roles for behaviour change communication in a changing malaria landscape. Therefore, the crucial role of these social and marketing strategies in improving availability, accessibility and promotion of AMFm-ACTs have been demonstrated [15].

At the time of this study, the effect of these AMFm supporting interventions on knowledge about clinical malaria diagnosis, prescribing by health care providers as well as on care and treatment of febrile illnesses was limited in Ghana. Previously, behaviour change communications in malaria have been done mainly through radio and television in Ghana. This study therefore directly targeted communities using different strategies from the existing ones in evaluating the effectiveness of AMFm activities on malaria management at the community level in rural settings.

## Methods

### Study design

Two rural districts the Asante-Akim North and South Districts of the Ashanti Region, Ghana were selected for the evaluation. Repeated cross-sectional pre and post surveys were deployed. This was a sub-component of a larger study that sought to improve malaria management practices in rural Ghana. The full details on the initial report and study settings are described in [16]. Asante-Akim North was the intervention site while Asante-Akim South District was a non-intervention comparison.

### Intervention

The focus of this intervention was to train community health workers, community based volunteers, and LCS attendants to promote effective counselling of healthcare workers and caregivers on proper administration of ACTs.

A series of consultations were arranged with the NMCP/GHS at the regional level to design and develop messages for LCS on malaria case management and proper administration of ACTs. Subsequently, meetings were held with leaders of LCS Associations to collate other messages on malaria case management and ACTs received from relevant state institutions such as the Pharmacy Council and Ghana Health Service (GHS) as well as non-governmental organizations (NGOs). Other information gathered included the difficulties LCS encountered in the acquisition and the sale of ACTs.

**Fig. 1:**
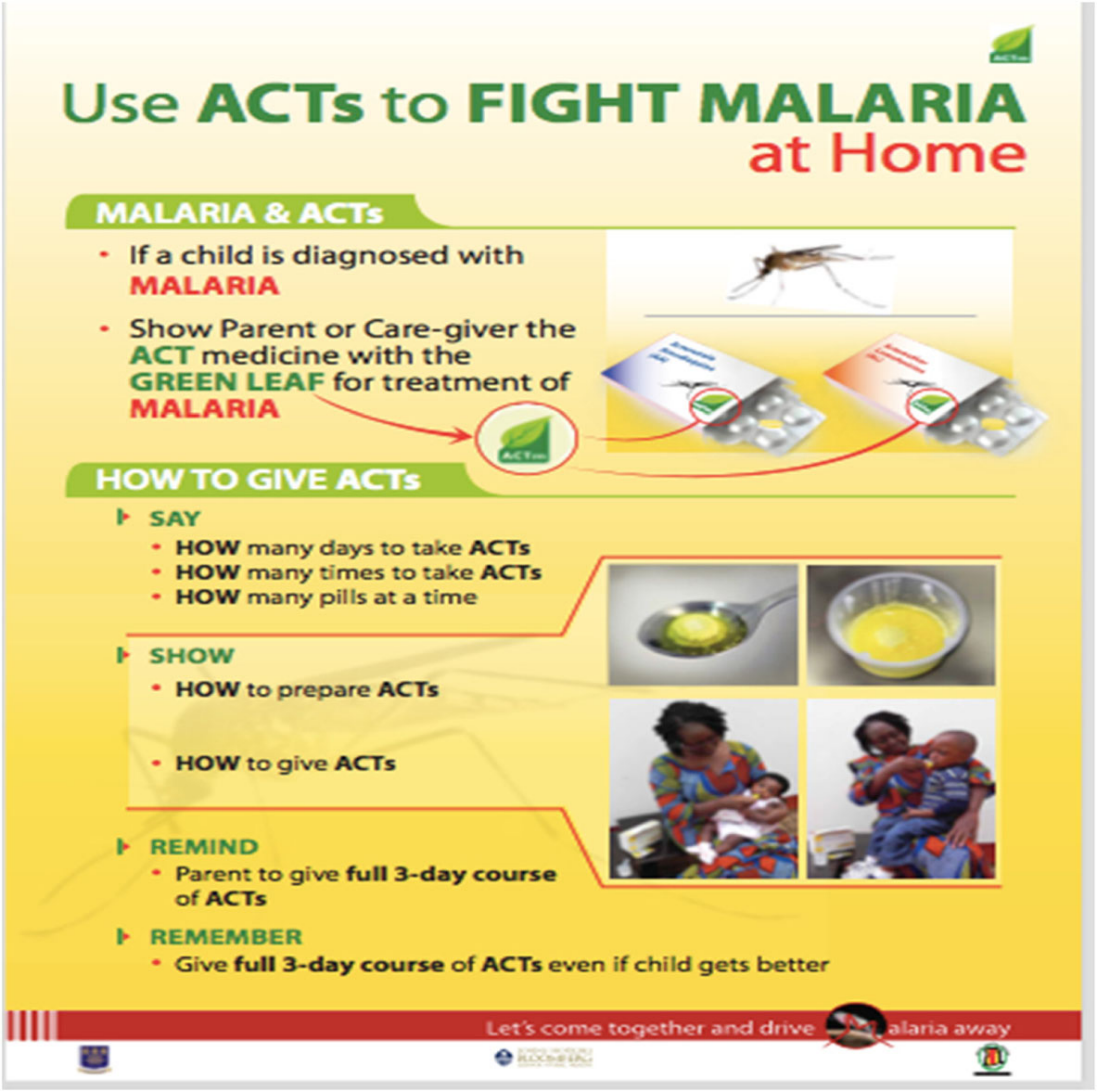
CHAI ACTm poster on malaria management

Communication materials including posters and flipcharts were developed to be used by CHW, LCS, and prescribers (Physicians, Physician assistants, and Nurses) in consulting rooms. This poster (Fig. 1) and Flipchart were pre-tested and demonstrated in communities by a local NGO. Posters were placed in all LCS shops in the Asante-Akim North District. Churches, Mosques, schools, youth groups, women associations, artisanal shops, and vehicle terminals were visited by trained staff of the local NGO. CHWs visited households and sensitized community members on individual basis. The effectiveness of the intervention was evaluated two months after implementation by conducting a post-intervention survey from randomly selected households.

### Ethical consideration

The study obtained approval from the Ghana Health Service Ethical Review Committee (GHS-ERC:10/7/11), the University of Ghana School of Medicine and Dentistry Ethical and Protocol Review Committee (MS-Et/M.11-P.5.3/2010–11) and the Johns Hopkins Bloomberg School of Public Health Institutional Review Board, Maryland, USA (IRB 3482). Informed consent was obtained from all study participants.

### Statistical analysis

**Fig. 2:**
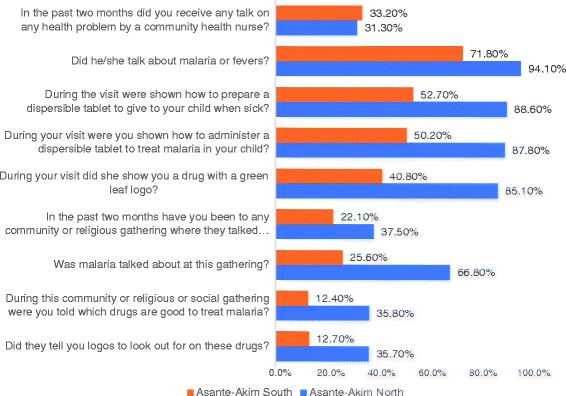
Final Household Survey: Exposure to Community Intervention Activities by CHOs/CBSV

**Fig. 3:**
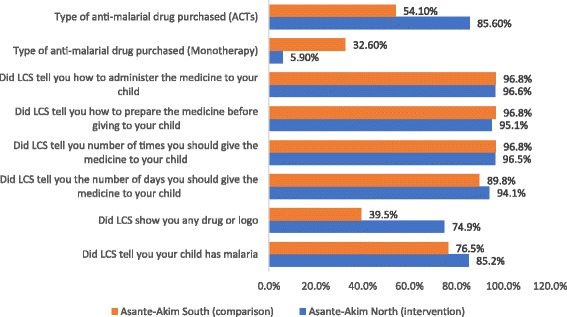
LCS information given to caregivers on anti-malarials purchased for children

**Fig. 4:**
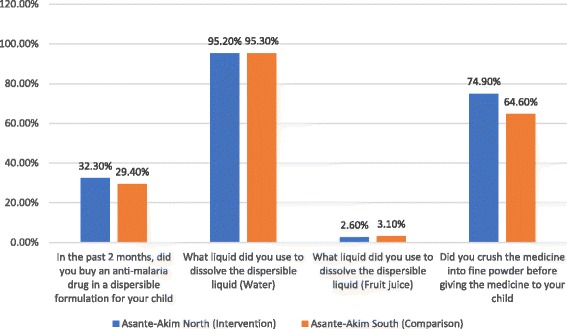
Final Household Survey: Preparation and administration of dispersible anti-malarial drugs

Data analysis included descriptive statistics (means, medians, frequencies, etc.) as well as comparison of study variables. A Univariate analysis produced descriptive statistics presented as proportions. Further evaluations of these associations were done using multivariate logistic regression technique, where odds ratios (OR) were computed. This necessitated recoding of both the predictor variables and the outcome variables. All the outcome variables were computed into composite variables that encapsulated the individual variables presented in Figs. 2, 3, 4 and Table 2. The composite, values were defined into binary variables. This procedure was followed for all the outcome variables produced by the study. For instance, in the outcome variable, exposure, all the individual variables were first recoded into 0 and 1 where 0 = No and 1 = Yes; thus, the computed exposure variable produced values with a minimum 0 and a maximum of 9. This was further dichotomized into exposed and non-exposed with exposed being 5 and above and non-exposed being below 5. In the second outcome variable (knowledge), the variable “What medicine will you recommend to a family member to give his/her child if the child had malaria?” was first recoded into ACT = 1 and non-ACT = 0. Similarly, “What logo do you look for?” was also recoded into AMFm logo = 1 and non-AMFm logo = 0 and when buying an anti-malaria drug, “do you look for any logo on the package of the medicine?” was also recoded into yes = 1 and no = 0. The variables measuring knowledge were three, therefore the range was between 0 and 3. This was further processed into below 2 being “less knowledgeable” and greater or equal to 2 being “knowledgeable”. The outcome variable (LCS information) was also recoded into binary variable where less than 4 = less information and 4 and above = adequate information on the range 0–7. All composite variables were coded Yes = 1 and No = 0. The outcome variable “dispersible preparation” was coded binary for the variable “what liquid did you use to dissolve the medicine”; with water/fruit juice = 1 and other = 0. All covariates were coded into categorical variables.

All analyses were performed using SPSS Version 20.0 (SPSS Inc. Chicago). *P*-value less than 0.05 was considered statistically significant.

## Results

A total of 1365 randomly sampled households participated in the post intervention household survey. Therewere 706 (51.7%) and 659 (48.3%) respondents from Asante-Akim North and South Districts respectively.

About 50% of the respondents sampled were in the age group of 26–35 years for both intervention and comparison Districts. Majority of the respondents 83.3% were females with more female participation from the intervention district as against the comparison group. About 45% of the total respondents have received secondary education and 38.1% had received primary level of education. More than 80% of the respondents in the intervention district were caregivers as against 60% in the comparison district. Details of the characteristics of the caregivers are shown in Table 1.

**Table 1:** Characteristics of respondents who purchased anti-malarial for their children

Characteristics of population sampled in the study districts			
	Asante-Akim North Frequency (%)	Asante-Akim South Frequency (%)	*p*-value
	*N* = 706	*N* = 659	
Age of Study Participant			
18–25	163 (23.1)	218 (33.1)	<0.001
26–35	360 (51.0)	299 (45.4)	
36–45	126 (17.8)	91 (13.8)	
Above 45	57 (8.1)	51 (7.7)	
Gender of parent/caretaker			
Male	92 (13.0)	136 (20.6)	<0.001
Female	614 (87.0)	523 (79.4)	
Education			
Primary	230 (32.5)	291 (44.2)	<0.001
Secondary	377 (53.4)	279 (42.4)	
Tertiary	48 (6.8)	40 (6.1)	
None	51 (7.3)	49 (7.4)	
Characteristics and symptoms of children whose parents/guardians purchased anti-malarials			
	Asante-Akim North	Asante-Akim South	*p*-value
Age of child	*N* = 590	*N* = 496	
Under 5 years	561 (95.1)	425 (85.7)	<0.001
5 and Above	29 (4.9)	71 (14.3)	
Sex of the child	*N* = 610	*N* = 505	
Male	316 (51.8)	215 (42.6)	<0.01
Female	294 (48.2)	290 (57.4)	
Sick within past 2 weeks	*N* = 572	*N* = 461	
Yes	242 (42.3)	214 (46.4)	0.186
No	330 (57.7)	247 (53.6)	
Fever within past 2 weeks	*N* = 572	*N* = 454	
Yes	240 (42.0)	226 (49.8)	<0.01
No	332 (58.0)	228 (50.2)	

There was a total of 1086 children in the households sampled, out of which majority 90.4% were under five years of age. With females constituting 52.8%. About 44.4% of the children sampled in the intervention district reported sick in the past two weeks. There were more fevers reported within past two weeks in the comparison district 49.8% than in the intervention district 42.0% as shown in Table 1.

About 31.3% and 33.2% of respondents in the intervention and comparison districts respectively said they had received a talk on a health problem by a community health nurse. There was no difference between reception of a health talk by a community health nurse (*p* = 0.446). Majority 94.1% of respondents from the intervention district and 71.8% from the comparison district also indicated that the talk was about malaria or fevers (*p* < 0.001).

Moreover, it was found that during the talk, 88.6% of respondents in the intervention district were shown how to prepare dispersible tablets for their children compared with 52.7% in the non-intervention district with a statistically significant difference at *p* < 0.001. Similarly, 87.8% and 50.2% agreed they were shown how to administer a dispersible tablet to treat malaria in the intervention and comparison districts respectively (*p* < 0.001). Again, when asked which logo was shown during the visit, 85.1% in the intervention district as against 40.8% in the comparison district said a drug with a green leaf logo was shown (*p* < 0.001). About 60% and 35.4% said a drug with a logo of a mosquito was shown in the intervention and comparison district respectively (Fig. 1).

The intervention activity involved religious, community and social groups educating people about malaria and its treatment using ACTs with the green leaf, this advocacy was towards eradicating monotherapies and promoting effective malaria treatment using the AMFm drugs. Details of the post-intervention survey are shown as in Fig. 2.

On the exposure to community intervention activities, the intervention site recorded 37.5% respondents who said they had been to a community/religious gathering where there was a talk on health problems while the comparison site reported 22.1% (*p* < 0.001). At these gatherings at the intervention site, about three-quarters (66.8%) of the respondents compared to 25.6% in the comparison district said malaria was talked about (*p* < 0.001). Furthermore, 35.8% of respondents at the intervention site mentioned that they were told which drugs are good to treat malaria compared to 12.4% at the comparison district (*p* < 0.001; Fig. 2).

The logos to look out for when purchasing a drug was a significant part of the intervention, when this was surveyed, 35.7% of the respondents from the intervention district said they were informed which logo to look out for when buying malaria drug whereas 12.7% of the comparison district respondents also said same. The difference in proportion for the two districts is significant (*p* < 0.001).

When respondents were asked what medicine, they will recommend to a family member to give his/her child if the child had malaria, 69.1% of the study participants in the intervention district said they would recommend ACTs (AL/AA) with majority mentioning (AA or AS/AQ) compared with 51.2% in the comparison district. The difference in proportion between the two districts is significant, *p* < 0.001 (see Table 2). Moreover, majority of respondents buying anti-malarial drug look for logo on the package. For this, close to 69% looked out for the Green Leaf Logo in the intervention district as against 41% from the comparison district (*p* < 0.001). However, other logos respondents looked out for were mosquito, mosquito in target, among other logos of local herbal medicines.

**Table 2:** Caregivers knowledge on anti-malarials and malaria management

	Asante-Akim North Frequency (%)	Asante-Akim South Frequency (%)	*p*-value
What medicine will you recommend to a family member to give his/her child if the child had malaria?	*N* = 680	*N* = 613	
-Monotherapy	117(17.3)	186(30.4)	<0.001
-ACTs	470(69.1)	314(51.2)	
-Other	93(13.6)	113(18.4)	
When buying an anti-malaria drug, do you look for any logo on the package of the medicine?	*N* = 706	*N* = 659	
Yes	484(68.6)	318(48.3)	*p* < 0.001
No	222(31.4)	341(51.7)	
What logo do you look for?	*N* = 484	*N* = 318	
AMFm green leaf logo	330(68.2)	131(41.2)	*p* < 0.001
Mosquito	98(20.2)	165(51.9)	
Mosquito in a target	22(4.5)	10(3.1)	
Others	34(15.1)	12(3.8)	

Respondents were asked to indicate whether within the last 2 months they had visited or sent someone to the Licensed Chemical Seller’s shop due to fever of their wards; less than half of the respondents (36.3%) and (31.6%) answered in the affirmative for the intervention and comparison districts respectively (*p* = 0.067). Out of those who visited, majority of the respondents in both the intervention and comparison districts, 76.1% and 73.4% respectively, bought anti-malarial drugs. With respect to the type of anti-malarial drugs purchased, 85.6% bought ACTs in the intervention district compared with 54.1% in the comparison district (*p* < 0.001). Similarly, about one-third (32.6%) of respondents in the comparison district purchased an antimalarial drug that was monotherapy compared with only 5.9% in the intervention district (*p* < 0.01). Specific anti-malarial drugs that were bought in both districts included Amodiaquine, Artesunate-Amodiaquine (AA), Artemether-Lumefantrine, herbal medicines, with AA being the most purchased drug in both districts (74% in intervention district and 33.3% in comparison district). No respondent bought chloroquine in the intervention district but close to 1% of respondents bought chloroquine in the comparison district.

When asked whether the LCS told them that their wards had malaria, 85.2% and 76.5% of respondents in both the intervention and the comparison districts respectively said they were told (*p* = 0.034). Fifty-two percent (52%) of respondents in the intervention district said the LCS showed them a mosquito logo and 38.7% of respondents in the comparison district were shown a mosquito logo. With respect to whether respondents were shown a green leaf logo, a large percentage of respondents in the intervention district (74.9%) said the LCS showed them a green leaf logo but only 39.5% of respondents in the comparison district said LCS showed them a green leaf logo (*p* < 0.001) (see Fig. 3).

About 94% and close to 90% of the respondents in the intervention and comparison district respectively said they were told the number of days they should give the medicine to the child (*p* = 0.224). Almost all the respondents said they were told how many times they should give the medicine to their child (96.5% and 96.8%, *p* = 0.892), how many tablets they should give (96.5% and 92.4%), how to prepare the medicine before giving to the child (95.1% and 96.8%, *p* = 0.412) and lastly how to administer the medicine to the child (96.6% and 96.8%, *p* = 0.881) all in the intervention and comparison districts respectively as illustrated in Fig. 3.

Preparation of the anti-malarial drug in dispersible formulation was also surveyed and these were the responses elicited; for the number of people who purchased anti-malarial drug in the dispersible formulation, the intervention district recorded 32.3% respondents who answered in the affirmative whereas 29.4% respondents in the comparison site did same (*p* = 0.254). For the people who purchased dispersible in the past two months, 95.2% of the intervention district respondents prepared the drug with water 95.3% of the people from the comparison did same. But for fruit juice as a liquid used to prepare the dispersible drug, the intervention district recorded 2.6% while the comparison site recorded 3.1%. During the preparation, almost three-quarter (74.9%) of the respondents in the intervention district crushed the medicine into fine powder before administering to the child whereas only about two-third (64.6%), did so in the non-intervention district (*p* = 0.023).

**Fig. 5:**
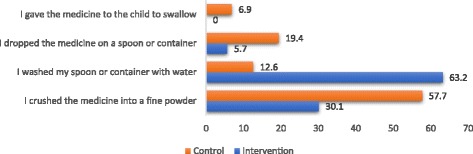
First step taken to prepare the dispersible for child

Concerning how to prepare a dispersible, caregivers were asked of the first step they went through in preparing the drug for the child, and these were the responses; more than half (57.7%) of the caregivers in the comparison site crushed the medicine into fine powder whereas 30.1% of caregivers in the intervention district did this. Approximately, 63% of the caregivers from the intervention site initially washed the spoon or container before drug administration as compared with 12.6% caregivers from the non-intervention site. About one-fifth (19.4%) respondents from the comparison district dropped the medicine on a spoon or container as compared to 5.7% in the intervention site. None of the respondents from the intervention site gave the medicine to the child to swallow whereas 6.9% of comparison site respondents did that (see Fig. 5).

Comparing the two districts, participants from the Asante Akim North district were more likely to be exposed to malaria/ACTm intervention by CHOs and CBSV (OR = 16.02; 95% CI = 7.88–32.55). All other predictors did not seem to have influenced exposure to the interventions as *p*-values were >0.05. Similarly, participants from the Asante-Akim North district were more knowledgeable about malaria management compared to their counterparts from the Asante-Akim South district (OR = 3.63; 95% CI = 2.52–5.23). All other predictors did not show any statistical significance.

It is noteworthy, that the same predictors did not produce a good fit model with LCS information and dispersible administration as outcome variables. All the predictors were statistically insignificant and therefore could not influence the model. However, the results indicate females had increased likelihood of preparing dispersible with water or fruit juice compared to males (OR = 4.78; 95%CI = 1.02–22.22).

## Discussion

This study was a part of a larger study conducted to gain further insight into AMFm implementation activities in rural Ghana. In rural settings where distance, cost and infrastructural challenges limit access to expert services, bringing treatment closer to the community is considered an attractive option to improving access to care [17]. This component of the study, therefore, sought to evaluate the effectiveness of AMFm implementation-related intervention specifically on malaria management practices in these hard-to-reach areas.

The data indicated that antimalarial drugs were purchased for children under the age of five years in both the intervention and non-intervention districts, and that, caregivers in both districts expressed a willingness to purchase ACT for treatment of malaria for children. This is consistent with findings from previous studies that reported that ACT uptake is facilitated by a communications campaign [18], and with findings from studies demonstrating that caregivers of sick children are more likely to purchase an ACT compared to caregivers of adults [19].

The data also showed that ACT is more likely to be purchased, and the AMFm logo is a key factor on taking decisions on antimalarial drug purchase in the intervention district. It is plausible to assume that implementation of the multi-pronged behaviour change communication strategies in the intervention district was a major contributory factor to the observed findings. In this respect and consistent with findings from previous studies, malaria prevention and treatment behaviours have been shown to be improved by appropriate evidence-based, and theory-driven social and behavioural change communication strategies in general [20], and combination of behavioural change communication interventions have been shown to be more specifically effective [14]. Communication campaigns based on interpersonal communications have also been demonstrated to improve demand for quality antimalarials, and improves adherence to drug treatment [13, 21]. Thus, it can be assumed that the difference in exposure to the suite of interventions by CHOs/CBSV between the two districts is the most likely explanation for the improved response to antimalarial management practices in the intervention district. These conclusions are further corroborated by the findings showing that while LCS provided adequate information on drug preparation and administration procedures in both intervention and non-intervention districts, LCS were more likely to demonstrate correct preparation and administration procedures for dispersible ACT in the intervention district. This is further supported by the multivariate results which indicate the impact of the intervention with respect to the exposure and knowledge (see Table 3). The finding that caregivers in the non-intervention district were more likely to purchase or to recommend antimalarial monotherapy is to be expected, and could be ascribed in part, to perceived (probably financial) barriers to ACT access [22]; however, importantly this finding highlights the need to engage the full spectrum of stakeholders in planning intervention activities and programs.

**Table 3:** Predictors of exposure to malaria and ACTm intervention and knowledge on malaria management

	Exposure		Knowledge	
Predictors	OR	95% CI	OR	95% CI
District				
Asante Akim North	16.02***	7.88–32.55	3.63***	2.52–5.23
Asante Akim South	*Ref*		*Ref*	
Gender				
Male	1.44 *Ref*	0.53–3.93	1.35 *Ref*	0.77–2.36
Female				
Age				
18–25	*Ref*		*Ref*	
26–35	1.07	0.31–3.73	1.85	0.91–3.75
36–45	1.18	0.35–3.97	1.58	0.81–3.07
Above 45	1.56	0.39–6.30	1.46	0.69–3.14
Education level				
None	*Ref*		*Ref*	
Primary	1.747	0.31–9.71	0.50	0.05–4.53
Secondary	1.351	0.43–4.30	0.72	0.09–6.05
Tertiary	0.667	0.21–2.13	0.55	0.07–4.61
Cox & Snell R Square	0.23		0.08	

***P-value <0.01

Ref - Reference for OR calculations

## Conclusion

Taken together, the findings from this study showed an improved knowledge and enhanced exposure to malaria management information and competencies among relevant stakeholders in the hard-to-reach intervention district. These outcomes were most likely a result of our deliberate implementation of intense social marketing and behavioral change communication strategies which promoted adherence to the subsidized ACT. In addition, our findings show that the availability of subsidized ACT coupled with the intensity of the communication campaigns have contributed to AMFm-related awareness in the study area. However, the availability of ACTs on the National Health Insurance Scheme (NHIS) could have influenced the outcome in terms of accessibility.

## Abbreviations

ACT: Artemisinin-based combination therapy (ACT)
AMFm: Affordable Medicine Facility-malaria
CBSV: Community based surveillance and volunteers
CHW: Community Health Workers
GHS: Ghana Health Service
IRS: Indoor Residual Spraying
LCS: Licensed Chemical Sellers
LLIN: long-lasting insecticide treated nets
NGO: Non-Governmental Organization
NHIS: National Health Insurance Scheme
NMCP: National Malaria Control Programme
OPD: Outpatient department
SP: Sulfadoxine-Pyrimethamine
